# Scanning Electrochemical Microscopy-Somatic Cell Count as a Method for Diagnosis of Bovine Mastitis

**DOI:** 10.3390/biology11040549

**Published:** 2022-04-01

**Authors:** Shigenobu Kasai, Ankush Prasad, Ryoma Kumagai, Keita Takanohashi

**Affiliations:** 1 Graduate Department of Electronics, Tohoku Institute of Technology, Sendai 982-8577, Japan; m181803@st.tohtech.ac.jp (R.K.); s1811242@st.tohtech.ac.jp (K.T.); 2 Department of Biophysics, Faculty of Science, Palacký University, 783 71 Olomouc, Czech Republic

**Keywords:** mastitis, bovines, cows, somatic cell count, respiratory activity

## Abstract

**Simple Summary:**

Mastitis is inflammation/swelling in the breast, which is generally caused by an infection. In this study, we present scanning electrochemical microscopy-somatic cell count (SECM-SCC) as a novel method for diagnosis of mastitis in bovines. We developed a biosensor in this study that can serve as a highly promising portable electrochemical device for mastitis diagnosis in bovines.

**Abstract:**

The method to diagnose mastitis is generally the somatic cell count (SCC) by flow cytometry measurement. When the number of somatic cells in raw milk is 2.0 × 10^5^ cells/mL or more, the condition is referred to as mastitis. In the current study, we created a milk cell chip that serves as an electrochemical method that can be easily produced and used utilizing scanning electrochemical microscopy (SECM). The microelectrode present in the cell chip scans, and the difference between the oxygen concentration near the milk cell chip and in bulk is measured as the oxygen (O_2_) reduction current. We estimated the relationship between respiratory activity and the number of somatic cells in raw milk as a calibration curve, using scanning electrochemical microscopy-somatic cell count (SECM-SCC). As a result, a clear correlation was shown in the range of 10^4^ cells/mL to 10^6^ cells/mL. The respiration rate (F) was estimated to be about 10–16 mol/s per somatic cell. We also followed the increase in oxygen consumption during the respiratory burst using differentiation inducer phorbol 12-myristate 13-acetate (PMA) as an early stage of mastitis, accompanied with an increase in immune cells, which showed similar results. In addition, we were able to discriminate between cattle with mastitis and without mastitis.

## 1. Introduction

Bovine mastitis leads to inflammation in the mammary glands [1,2,3]. It is caused by the invasion and proliferation of pathogenic microorganisms from the teat opening into the mammary gland [4]. Mastitis is said to be one of the largest problems in the dairy industry [3,5,6], and about 380,000 cases occur annually in Japan, accounting for about 30% of all dairy cow injuries. The detection and treatment of asymptomatic latent mastitis is crucial for reducing economic losses. It has been reported that the number of somatic cells in raw milk ranges somewhere between 1.0–2.5 × 10^5^ cells/ mL for categorizing or treating latent mastitis. Therefore, in this manuscript, the number of somatic cells in raw milk is taken as normal when it is less than 1.0 × 10^5^ cells/ mL and subclinical/latent mastitis when it is in the range of 1.0 × 10^5^ cells/ mL to 2 × 10^5^ cells/ mL. The value 2 × 10^5^ cells/ mL or more is treated as clinical mastitis. These limits are highly variable, depending on the geographical region. In most developed dairy industries, for instance, the European Union, directives have set a limit of 400,000 cells/mL for SCC in raw buffalo milk, while in United States, this limit is set to 750,000 cells/mL. Somatic cell count in cow milk >200,000 cells/mL indicates mastitis according to the directives of the International Dairy Federation [7,8].

It is also estimated to cause an annual economic loss of $2 billion in the United States [9]. In the early stages of mastitis, the number of leukocytes in raw milk (further referred to as milk in the text) increases to combat the bacteria that have invaded the mammary gland, and the number of somatic cells in milk increases to about 10^5^ cells/mL. As the condition progresses, cells on the surface of the mammary gland that have died due to inflammation are shed, and the number of somatic cells in milk increases to about 10^6^ cells/mL. The number of somatic cells in milk is used as an index for mastitis detection, and many testing methods have been developed [10,11,12,13,14,15]. Three typical somatic cell count evaluation methods are widely known. The first method, the California mastitis test (CMT) [16], is a method of visually observing and discriminating a gelled sample by destroying the cell membrane of somatic cells in milk and reacting DNA with a chemical reagent. This method is simple and does not require a device or complicated operation and is used as a simple inspection method in farms; however, it is difficult to distinguish between healthy bovines and early mastitis bovines because it is judged by colour and shape, and thus early detection is difficult [17]. The second method is a test method that measures the electrical conductivity of milk [18,19]. This method is rather simple and is incorporated in milking machines and milk parlours; however, the value changes due to environmental fluctuations such as temperature rather than cells. It is difficult to measure accurately using this method. The third method is a somatic cell count (SCC) method [20] that uses flow cytometry, which during the past decade has been widely used as a mastitis test method to directly count individual cells [3]. It is quantitative, while CMT and electrical conductivity are qualitative methods. By this method, the cattle are determined to have mastitis when the number of somatic cells in milk is 2.0 × 10^5^ cells/mL or more, and caution is required when the number of cells in milk ranges between 10^5^ cells/mL and 10^6^ cells/mL [21,22]. Since this measuring device is large and expensive, it is difficult to install in each parlour. In Japan, it is typical to send raw milk to a testing facility to perform this test, and thus real-time testing is difficult on a daily basis, making early detection challenging. In recent years, as a method for monitoring the increase in leukocytes in the early stage of mastitis for earlier detection, inflammatory cytokine evaluation [23,24], measurement of hydrogen peroxide (H_2_O_2_) using chemiluminescence method [25], etc., have been used. A method for evaluating superoxide anion radicals, which is one of the reactive oxygen species (ROS), was also developed [26]. However, these methods require expensive reagents such as antibodies and enzymes.

Aiming for the development of a bovine mastitis test method that can be easily tested by dairy farmers in real time, we decided to study an electrochemical method that can measure using a small amount of sample and inexpensive reagent. Scanning electrochemical microscopy (SECM) [27,28] using a microelectrode as a probe has been used for single-cell studies [29,30] and single-molecule level measurements in the past [31]. Since cells in body fluids and culture fluids consume oxygen during respiration, the dissolved oxygen concentration of the solution in the vicinity decreases, and a diffusion layer is formed due to the difference in concentration. The dissolved oxygen (DO) concentration in the solution can be measured as an oxygen reduction current by applying a voltage to the platinum microelectrode of the SECM probe. Using SECM, Shiku et al. scanned the microelectrode up and down in the Z-axis direction to evaluate the quality of fertilized bovine embryos by measuring respiratory activity [29]. Recently, Kaya et al. measured the correlation between the number of bacteria in collagen-embedded culture and respiration activity [32]. In addition, we also evaluated the respiration activity and respiratory bursts of human cells in our studies [33,34,35].

The current study examines whether it is possible to monitor the increase in leukocytes in early bovine mastitis through evaluation of respiratory activity. In addition, imaging the 3D distribution of respiration activity by SECM can be used as a simple test by a dairy farmer in real time. We first standardized the centrifugation conditions to avoid electrode adsorption by milk components, devised cell chipping and the hemispherical diffusion distribution by SECM, and examined whether there was a correlation between respiratory activity taking into account cell count and the number of somatic cells in milk. Furthermore, we created a calibration curve for measuring the number of somatic cells by evaluating the respiration activity of 10^4^ to 10^6^ cells/mL, which is necessary for bovine mastitis testing. The calibration curve needs to be extended with more data points and to reach saturation. In addition, we also investigated whether SECM-SCC can detect increased oxygen consumption under induced respiratory bursts specifically caused by immune cells such as monocytes and neutrophils. As an output, the current work led to the development of a new simple test method for bovine mastitis.

## 2. Materials and Methods

### 2.1. SECM-SCC with Inverted Cone-Shaped Well

**Figure 1:**
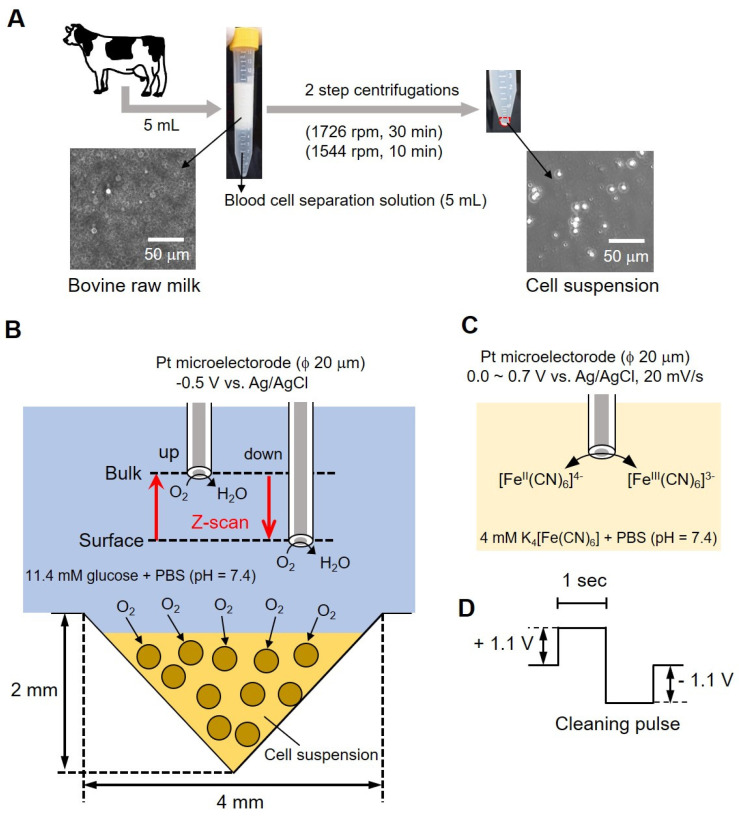
Conceptual diagram of bovine mastitis test methodology. Centrifugal method of the bovine milk (**A**); conceptual diagram of the evaluation of somatic cell respiration activity by measuring the oxygen reduction current value (**B**); reaction mechanism at the platinum electrode (**C**) and cleaning pulse (**D**).

Figure 1 shows a conceptual diagram of the simple bovine mastitis test method performed in this study. Milk collected from bovines in Figure 1A contained fat (4%) and protein (3%) in addition to somatic cells. Centrifugation was required (as shown in the photograph of the centrifuge tube in (A) to remove these components (fat and proteins). An equal volume (5 mL each) of blood cell separation solution was mixed with milk and centrifuged at 1726 rpm for 30 min, and the supernatant containing fats and protein (~9 mL) was removed. Furthermore, a second centrifugation (1544 rpm, 10 min) was done by adding 3 mL of 11.4 mM glucose-containing phosphate buffer saline (PBS) to remove the blood cell separation solution. The precipitate suspended in a small volume of buffer (cell-aggregated milk somatic cells) was then used for measurement. We took 10 μL of the sample on a hemocytometer and counted the number of cells. The samples were adjusted to 6.00 × 10^6^ cells/mL and 6.00 × 10^5^ cells/mL. The number of somatic cells in raw milk was measured using the hemocytometer to distinguish between the cells and fat globules. The photograph (Figure 1B, lower panel) shows a photomicrograph of milk before centrifugation, and the photograph on the right shows a photomicrograph of the cell suspension after centrifugation. Somatic cells with a diameter of about 10 to 20 μm can be observed.

For measurements, 8 μL of sample was taken in an inverted cone-shaped well (diameter (ϕ) 4 mm, depth 2 mm) and allowed to stand for 15 min. Following that, a platinum microelectrode (ϕ 20 μm) was used as the working electrode; Ag/AgCl was used as the counter electrode and reference electrode. A total of 10 mL of 11.4 mM glucose-containing PBS was used as the measurement solution. As presented in Figure 1C, we measured the difference in oxygen reduction current between surface and bulk by sweeping back and forth vertically (referred to as z-scan) while applying a voltage to the microelectrode. We evaluated the respiratory activity of cells from the difference in current value by scanning electrochemical microscopy-somatic cell count (SECM-SCC). The platinum microelectrode was held 20 μm above the cell suspension at an applied voltage of -0.5 V vs. Ag/AgCl and scanned 1000 μm in the Z direction while measuring the oxygen reduction current. The scan rate was kept at 10 μm/s; the number of scans was 3 and the sampling time was 100 ms. The microelectrode reciprocated between a position (surface) about 20 μm above the surface of the cone-shaped well and about 1020 μm (bulk) at intervals of 200 s.

### 2.2. Evaluation of Electrodes over Time: Validating Stability Using Cyclic Voltammetry Measurements

Cyclic voltammetry (CV) was performed to evaluate the change in the value of current over time to investigate the cause of the difficulty in measuring respiration activity at the above-mentioned cell count of 10^4^ cells/mL or less. The CV of the Pt microelectrode was measured using 4 mL of 4 mM potassium ferrocyanide solution as the measurement solution and Ag/AgCl as the counter electrode and reference electrode, respectively. We measured the oxygen reduction current at a sample-electrode distance of 20 μm. The CV of the Pt microelectrode at 1, 3, 5, and 10 min after the start of measurement was compared with the CV of the Pt microelectrode before measurement. In addition, to observe the change in the current value, a cleaning pulse (Figure 1D) was applied to the electrode, and cyclic voltammetry was performed again (with 12 repetitions). Somatic cells that had been centrifuged and removed from milk were mixed with collagen gel at a ratio of 1:9, and 8 μL was poured in an inverted cone-shaped well. A Pt microelectrode (ϕ 20 μm) was used as the working electrode, Ag/AgCl was used as the counter electrode and reference electrode, and 10 mL of 11.4 mM glucose-containing PBS was used as the measurement solution. The sample-electrode distance was fixed at 20 μm. In this state, the oxygen reduction current was measured for 10 min. Before and after this measurement, cyclic voltammetry of 0.0~0.7 V vs. Ag/AgCl at 20 mV/s was performed using 4 mM K_4_Fe(CN)_6_-containing PBS to evaluate the effect of the collagen embedding treatment.

### 2.3. Cell Biochip to Obtain the Sensitivity Required for SECM-SCC

**Figure 2:**
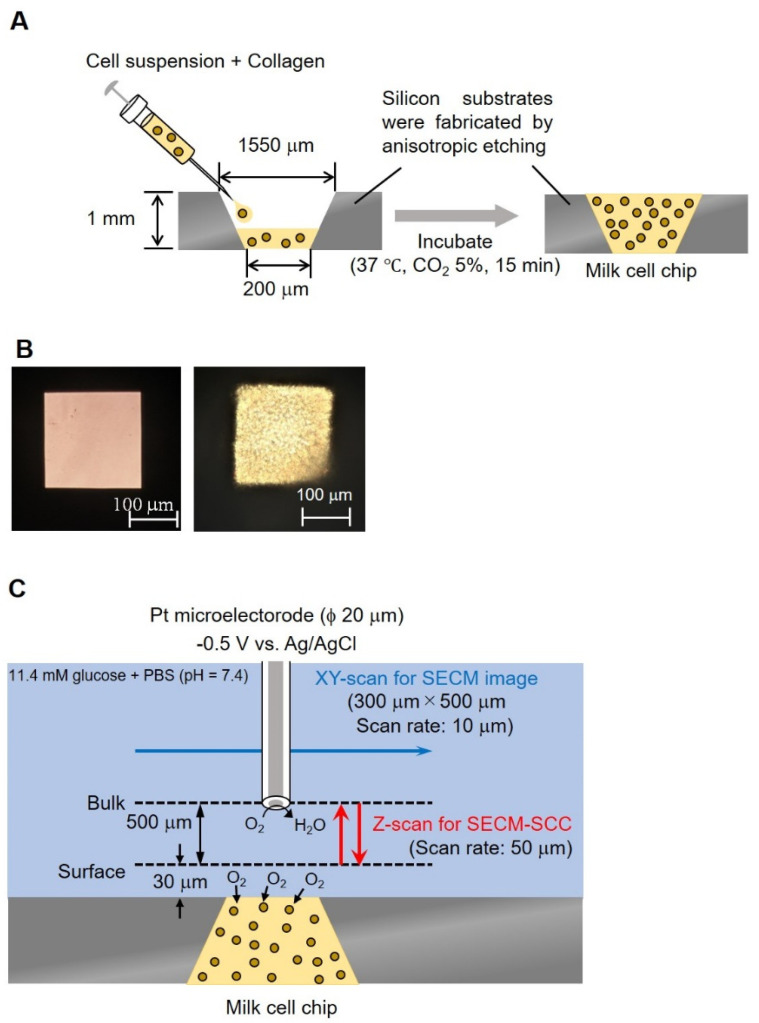
Conceptual diagram and photographs of milk cell chips. Schematic diagram of milk cell chips (**A**). Milk (−) and milk (+) (**B**). The experimental method of XY-scan and Z-scan (**C**). In the XY-scan, the distance between the microelectrode and the milk cell chip was set to 30 μm or 160 μm and scanned the range of 300 μm × 500 μm while applying −0.5 V vs. Ag/AgCl. In the Z-scan, −0.5 V vs. Ag/AgCl was applied to the Pt microelectrode. The working electrode was held 30 μm above the top of the well and scanned 500 μm in the Z direction to measure respiration activity. The scan rate was kept at 50 μm/s; the number of scans was 3 and the sampling time was 100 ms.

A silicon substrate having an inverted pyramid-shaped fine groove was produced by anisotropic etching, which is a three-dimensional microfabrication technique. The lower surface (200 × 200 μm) of this well should be in μm size that can be easily measured by SECM, and this surface is used as the detection port. In addition, the upper surface (1550 × 1550 μm) is in mm, making it easy for collagen insertion. Processed somatic cells taken from milk were mixed with collagen gel at a ratio of 1:9 and transferred (1.5 μL) in pyramid-shaped wells (1.4 cm, 3.6 cm, 1 mm well), lower surface (200 × 200 μm), upper surface (1550 × 1550 μm)) followed by incubation at 37 °C for 15 min (Figure 2A). Figure 2B shows a photograph of a well without and with somatic cells.

### 2.4. Understanding the Shape of the Oxygen Diffusion Layer

In order to understand the three-dimensional distribution of the oxygen concentration on the milk cell chip, the oxygen reduction currents in the XY-direction and the Z-direction were visualized while being held at the oxygen reduction potential. We used Pt microelectrodes (ϕ 20 μm) as the working electrode, Ag/AgCl as the counter electrode and reference electrode, and 15 mL of 11.4 mM glucose-containing PBS as the measurement solution. By keeping the distance between the upper surface of the well and the microelectrodes at 30 or 160 μm and scanning the vicinity of the upper surface of the well for 300 × 500 μm in the XY-direction, an image of the oxygen concentration distribution near the upper surface of the well was taken (Figure 2C). The scanning speed was 50 μm/s, and the resolution was 10 μm. The number of cells was kept at approximately 3.00 × 10^6^ cells/mL. To examine the minimum scanning range required for Z-scan, the accurate oxygen concentration distribution in the vertical direction was measured by scanning in the vertical direction. The microelectrode was fixed at a position 20 μm above the chip detection port. After the oxygen reduction current value stabilized, a 10 μm upward scan was done at a rate of 10 μm/s. This operation was repeated until the distance between the microelectrode and the chip reached 140 μm.

### 2.5. SECM-SCC with Milk Cell Chip

Based on the results of the analysis of the Z-scan’s scanning distance, the surface was determined to be 30 μm above the milk cell chip. In the mastitis test method, it is important to discriminate between 2 × 10^5^ cells/mL, which is the criterion for mastitis, and lower. We used Pt microelectrodes (ϕ 20 μm) as the working electrode, Ag/AgCl as the counter electrode and reference electrode, and 15 mL of 11.4 mM glucose-containing PBS as the measurement solution. We applied −0.5 V vs. Ag/AgCl to the Pt microelectrode. The microelectrode was held 30 μm above the top of the well and 500 μm in the Z-direction was scanned to measure respiratory activity. The sweep speed was kept at 50 μm/s, the number of scans was 3, and the sampling time was 100 ms (Figure 2C). The microelectrode reciprocated between a position (surface) about 30 μm above the surface of the milk cell chip and about 530 μm (bulk) at intervals of 20 s. This experiment was performed on three samples, with cell numbers of 1.10 × 10^6^, 5.50 × 10^5^, and 5.50 × 10^4^ cells/ mL. These samples were prepared by diluting 1.10 × 10^6^ cells/mL from the same cow with the measurement solution.

In order to reach a simplified methodology, we also evaluated if centrifugation can be replaced by an alternative method. By inserting nanofibers into a syringe and passing raw milk without centrifugation, results were obtained by performing SECM-SCC. This has the added advantage of overall shortening of the sample preparation time.

### 2.6. Evaluation of Respiratory Burst Using SECM-SCC

Raw milk containing 4.15 × 10^6^ cells/mL somatic cells determined using flow cytometry at Miyagi Prefectural Livestock Association was evaluated in the absence and presence of 20 nM phorbol 12-myristate 13-acetate (PMA). PMA was used to induce respiratory burst, and comparative measurements were done. Other measurement conditions for the electrochemical system of SECM-SCC are the same as described in Section 2.5.

## 3. Results and Discussion

### 3.1. Correlation between Somatic Cell Count and Respiration Activity Using Inverted Cone-Shaped Well

**Figure 3:**
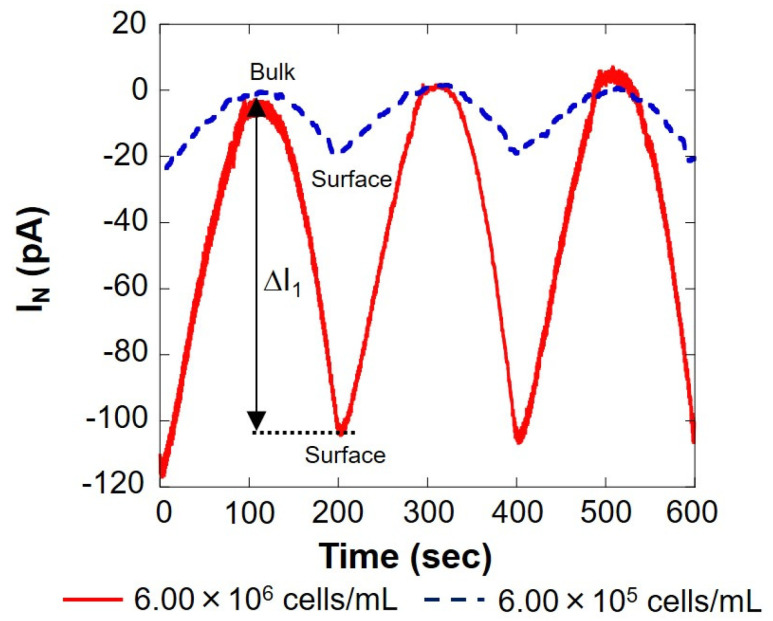
Changes in respiration activity with somatic cells. I_N_: relative value of oxygen reduction current in bulk, which was set at 0 nA.

The solid red line in Figure 3 shows the measurement result of 6.00 × 10^6^ cells/mL, and the blue dotted line shows the result of 6.00 × 10^5^ cells/mL, measured using the setup shown in Figure 1B. On the surface, the reduction current value was reduced by several tens of pA compared to the bulk, indicating that the dissolved oxygen concentration in the vicinity was reduced by the respiration of somatic cells. The current value at 0 s was about 20% higher than that at 200 and 400 s. This is because the scanning was started immediately after the voltage was applied to the working electrode, and the measurement was performed before the current value became stable. In this study, the value at 0 s was not used when calculating the current value difference (ΔI) between the bulk and the surface. As presented in Figure 3, the difference in oxygen reduction current value between the bulk and surface on the first cycle (round trip) |ΔI_1_| was 100 s when the distance between the microelectrode and the cell suspension was the largest and 200 s when it was the smallest. Similarly, the difference in oxygen reduction current value between the two points of the other two cycles (two round trips) were read, and the average |ΔI| was calculated. The difference in oxygen reduction current values was found to be about 105 pA at 6.00 × 10^6^ cells/mL and about 20 pA at 6.00 × 10^5^ cells/mL. The amount of dissolved oxygen in the measured solution at room temperature was about 248 μM, as measured in our laboratory. The oxygen reduction current value at this time was -1.76 nA. The difference in oxygen concentration (ΔC) was calculated from |ΔI| and is summarized in Table 1. 

Table 1 shows that there is a correlation between the number of somatic cells and the difference in oxygen concentration. From this result, it was possible to evaluate the number of somatic cells in milk by measuring respiratory activity. However, at the same time, it was difficult to measure respiratory activity with a cell count fewer than 10^5^ cells/mL.

### 3.2. Decrease in the Current Value over Time or Due to Collagen

**Figure 4:**
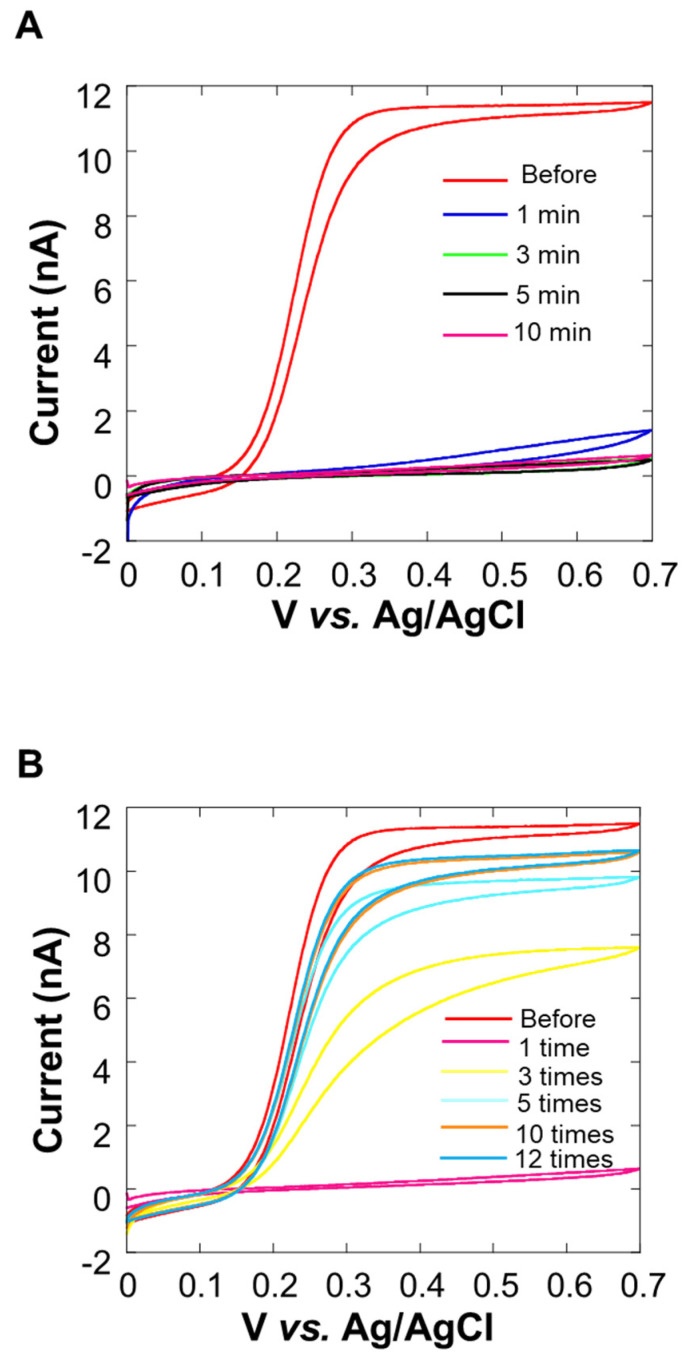
Cyclic voltammogram of 0.0~0.7 V vs. Ag/AgCl in potassium ferrocyanide solution at 0 min, 1 min, 3 min, 5 min, and 10 min after the measurement of the Pt microelectrode. Oxygen reduction current was measured at a sample-electrode distance of 20 μm.

In the cyclic voltammogram in Figure 4A, it can be observed that the current value decreases over time. After 10 min, the average current value at 0.5~0.7 V vs. Ag/AgCl was about 10% or less than it was before the experiment. We considered that this decrease in current value was the cause of insufficient sensitivity. In Figure 4B, it can be seen that the current value increases each time a cleaning pulse is applied (Figure 1D), and the waveform is close to sigmoidal. When the cleaning pulse was applied five times, the average current value at 0.5~0.7 V vs. Ag/AgCl was about 90% of the value observed before the experiment. However, the recovery of the current value was not seen when the number of times exceeded 10, and the value did not return to the original value before the experiment at the 12th time. For this reason, the appropriate use of cleaning pulses is effective for electrode activity.

**Figure 5:**
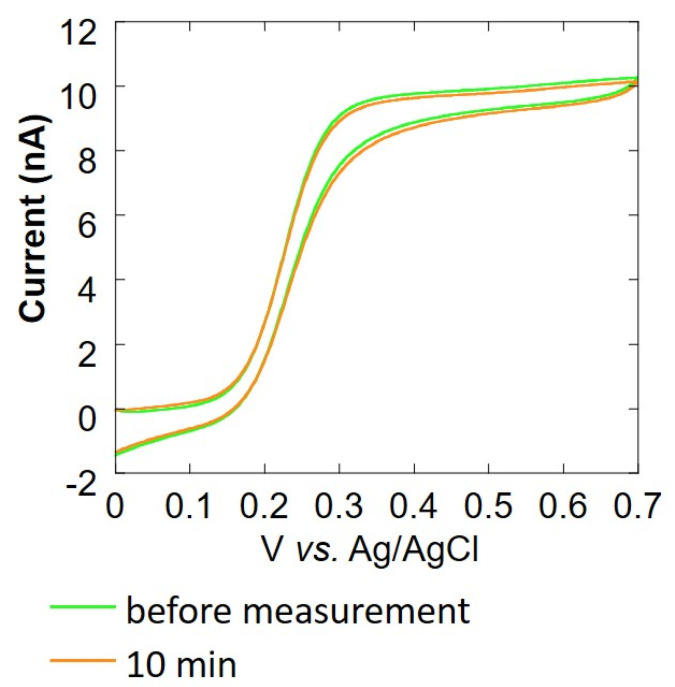
Evaluation of changes over time (in current value) by cyclic voltammetry upon collagen embedding.

Based on the experimental results over time by CV, it is considered that the adsorption of milk components on the electrode surface was the reason, so a method for suppressing the diffusion of milk components is required. We decided to embed cells using collagen [36] (Figure 2A), which is a biomaterial that inhibits the diffusion of milk components, and investigate the effect of suppressing the diffusion of milk components. Figure 5 shows the cyclic voltammogram before and after 10 min of the experiment. The average current value at 0.5~0.7 V vs. Ag/AgCl is about 99% of the current value before the experiment, and there is almost no change in the current value. From this, it is considered that the collagen embedding treatment can prevent the current value from decreasing over time.

### 3.3. Hemispherical Oxygen Diffusion Layer on Milk Cell Chip

**Figure 6:**
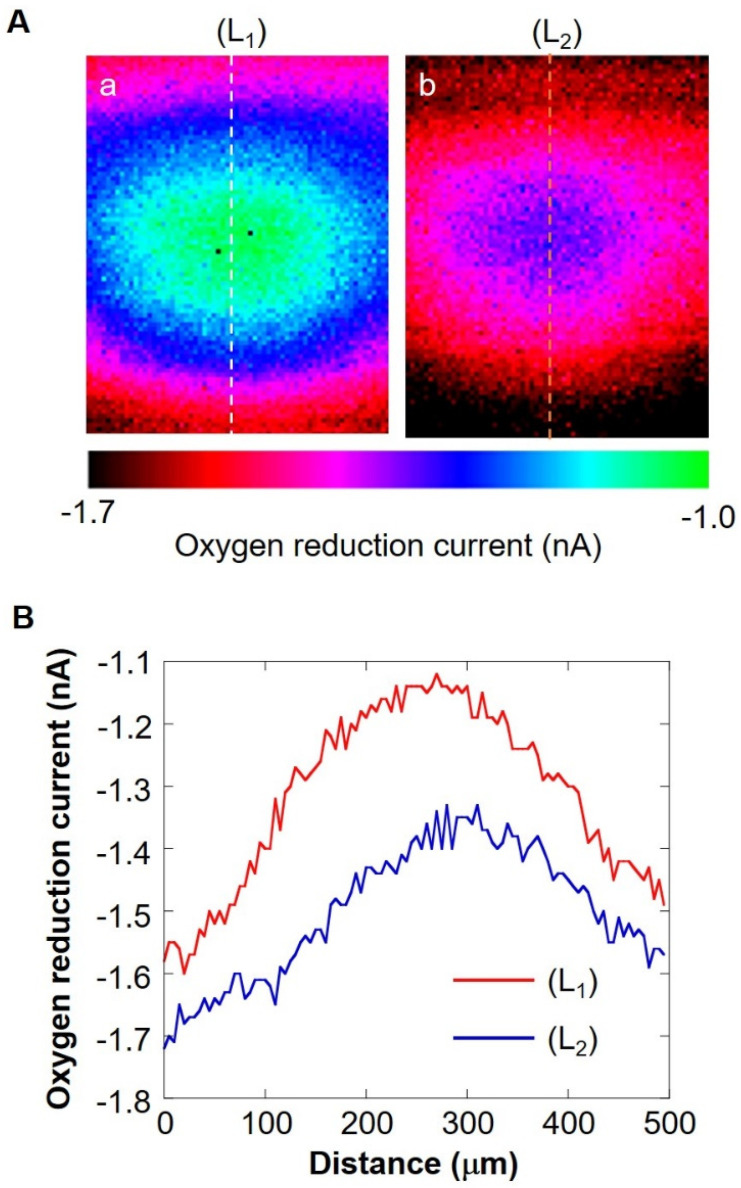
(**A**) XY-scan images showing the oxygen concentration distribution with the distance between Pt microelectrode and the cell chip of 30 μm (**a**) and 160 μm (**b**). Line (L_1_) and (L_2_) are on the same X coordinate. Graph (**B**) shows the oxygen reduction current values of line (L_1_) and line (L_2_).

**Figure 7:**
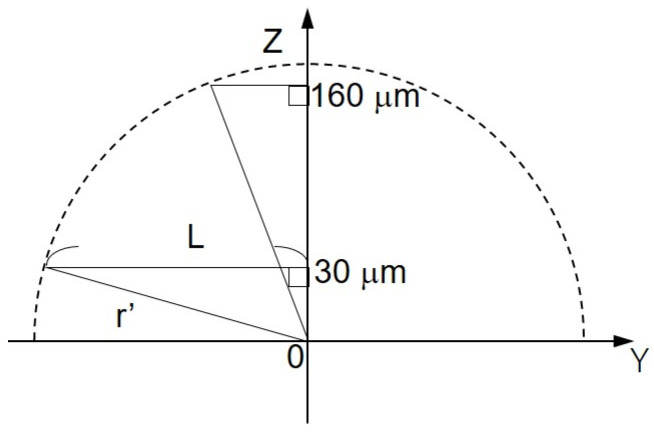
Schematic diagram of the oxygen concentration distribution on ZY plane. L, half the distance of the oxygen reduction current less than or equal to −1.4 nA; Z’, the distance between Pt microelectrode and the cell chip; r’, oblique side of the right triangle.

The shape of the oxygen diffusion layer was estimated by taking two XY images on the milk cell chip and comparing them. In Figure 6A(a,b), the oxygen reduction current values are distributed almost in a circle. It is suggested that the cause of the distortion in the x-direction is that the oxygen diffusion layer on the chip is disturbed in the x-direction because the microelectrodes are scanned in the x-axis direction. The line segments (L_1_) and (L_2_) exist on the same x-coordinate. We set L as half the length at which the oxygen reduction currents of the line segments (L_1_) and (L_2_) are -1.4 nA or less. We calculated r’ using the three-square theorem, and both were about 1.6 × 10^2^ μm. This result suggests that the oxygen concentration distribution exists as shown in Figure 7. These results show that a hemispherical oxygen diffusion layer is formed on the milk cell chip. Therefore, oxygen diffusion is considered to follow the spherical diffusion theory.

Since the oxygen diffusion layer on the milk cell chip is hemispherical, we considered f as the oxygen diffusion flux (mol/cm^2^), D as the oxygen diffusion coefficient (2.18 × 10^−5^ cm^2^/s), and C as the oxygen concentration of the surface (mol/cm^3^). C* is the bulk oxygen concentration (mol/cm^3^), r_s_ is the detection port radius (mm), and F is the oxygen consumption rate (mol/s). We set R = r + r_s_. The oxygen diffusion layer follows the hemispherical diffusion theory, and the initial conditions and boundary conditions are determined as follows:

t = 0, R ≧ r_s_ (r ≧ 0), C = C*

t ≧ 0, R → ∞, C = C*

t ≦ 0, R = r_s_ (r = 0), C = Cs

Then, the overall respiration rate F is

F = 2πr_s_D(C* − C_s_) (1)

**Figure 8:**
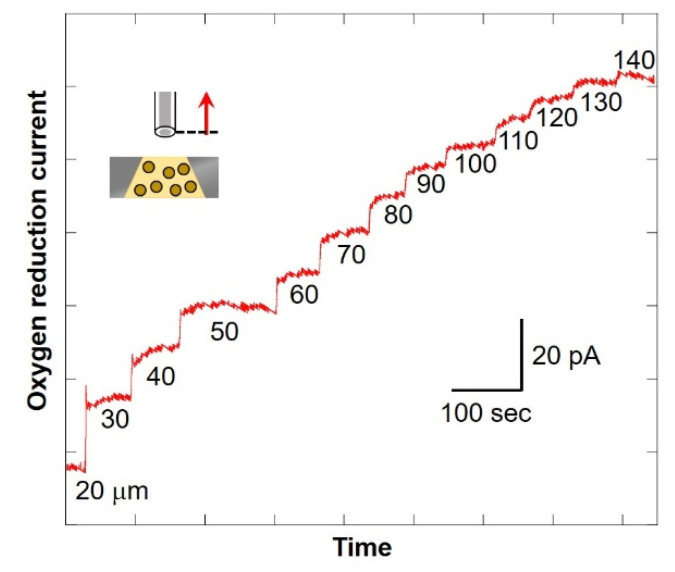
Changes in oxygen reduction current value in the Z-axis direction.

The numerical value at the bottom of the graph in Figure 8 shows the distance between the microelectrode and the chip (as shown in Figure 2C). After scanning, the current value was found to be increasing. In addition, the larger the distance between the microelectrode and the chip, the smaller the difference in value of current. This suggests that the oxygen consumption of the cells in the chip reduces the oxygen concentration near the detection port and forms a diffusion layer. Since there is almost no difference in current value when scanning from 130 μm to 140 μm, it is possible to compare the oxygen reduction current values at the point near the detection port and the point where the distance between the microelectrode and the chip is 140 μm or more. It is considered that oxygen consumption of somatic cells in milk can be measured. In this study, the scanning range was set as 30 μm to 530 μm.

### 3.4. SECM-SCC and Calibration Curve Using Milk Cell Chip

**Figure 9:**
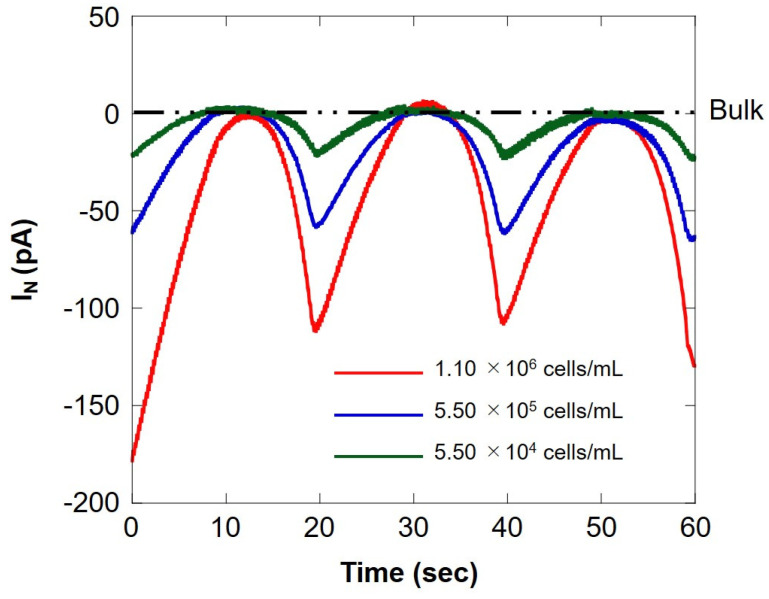
Comparison of the oxygen reduction currents measured via Z-scanning at three different cell concentrations. The red, blue, and green lines indicate the respiratory activity at a cell concentration of 1.10 × 10^6^ cells/mL, 5.50 × 10^5^ cells/ mL, and 5.50 × 10^4^ cells/mL, respectively. I_N_: relative value of oxygen reduction current in bulk, which was set at 0 nA.

We compared the respiratory activity of milk cell chips by cell number and examined the correlation with somatic cell number. Figure 9 shows SECM-SCC at three different cell concentrations. The red line, blue line, and green line show the measurement results for the difference in the number of cells of 1.10 × 10^6^ cells/mL, 5.50 × 10^5^ cells/mL, and 5.50 × 10^4^ cells/mL, respectively. When the microelectrode is located near the milk cell chip (surface) at 20 s intervals, an oxygen reduction current depending on the number of cells is obtained. In addition, it can be seen that it does not depend on the number of cells at the bulk position. This suggests that SECM-SCC using milk cell chips with collagen gel may measure in the range of 10^4^ cells/mL to 10^6^ cells/mL. Dissolved oxygen was set at 25 °C (248 μM), the oxygen reduction current value (−1.7 nA) was set, and the respiration rate (F) was calculated by the concentration and Equation (1) in the same manner as in Section 3.1; the results are summarized in Table 2. The value of respiration rate calculated from the hemispherical diffusion equation reflects the respiratory activity of somatic cells. Therefore, it is suggested that the oxygen reduction current value depends on the number of somatic cells.

**Figure 10:**
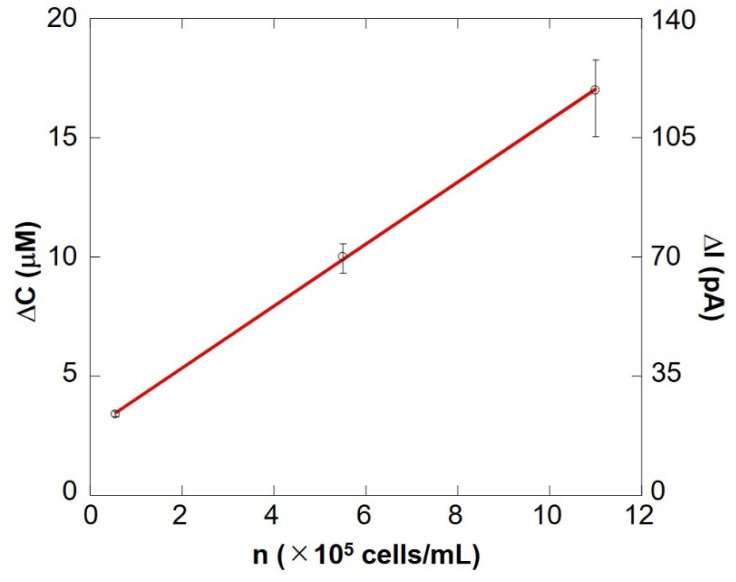
SECM-SCC calibration curve showing cell concentration vs. the change in oxygen concentration. The bar shows the standard deviation of each ΔI value obtained from multiple scans of SECM-SCC (*n* = 3).

Figure 10 shows a preliminary calibration curve for measuring the number of somatic cells by evaluating respiratory activity. The oxygen reduction current value linearly depends on the number of cells between 10^4^ cells/mL and 10^6^ cells/mL. From this result, it is evident that the number of somatic cells can be measured by evaluating the respiratory activity using an electrochemical method. The results show that the difference in respiration activity is due to the number of cells. SECM-SCC thus can be claimed to have sufficient sensitivity. In addition, it was possible to measure oxygen consumption in the same way as in Figure 9, with raw milk cell chips that were filtered using nanofibers instead of centrifugation to separate fat (Supplementary Figure S1).

### 3.5. Immune Cell Evaluation under Respiratory Burst Using SECM-SCC

**Figure 11:**
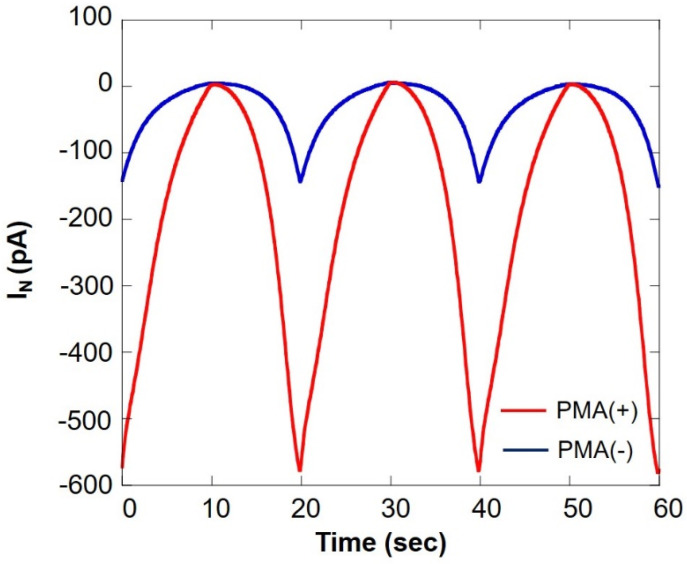
Comparison of oxygen consumption of somatic cells in milk cell chip using SECM-SCC during ordinary respiration (blue line) and during respiratory burst (red line) I_N_: relative value of oxygen reduction current in bulk, which was set at 0 nA.

Figure 11 shows a comparison of oxygen consumption of somatic cells in the milk cell chip using SECM-SCC during normal respiration and respiratory burst. It can be seen that the current value under PMA is increased about five times. This is because of the fact that PMA induces respiratory burst in immune cells such as monocytes and neutrophils. From these results, it is evident that the respiratory burst evaluation method using PMA in combination with SECM-SCC to specifically evaluate the immune cells in raw milk can be used for detection of latent breast inflammation and early stages of mastitis.

### 3.6. Bovine Examination by SECM–SCC

**Figure 12:**
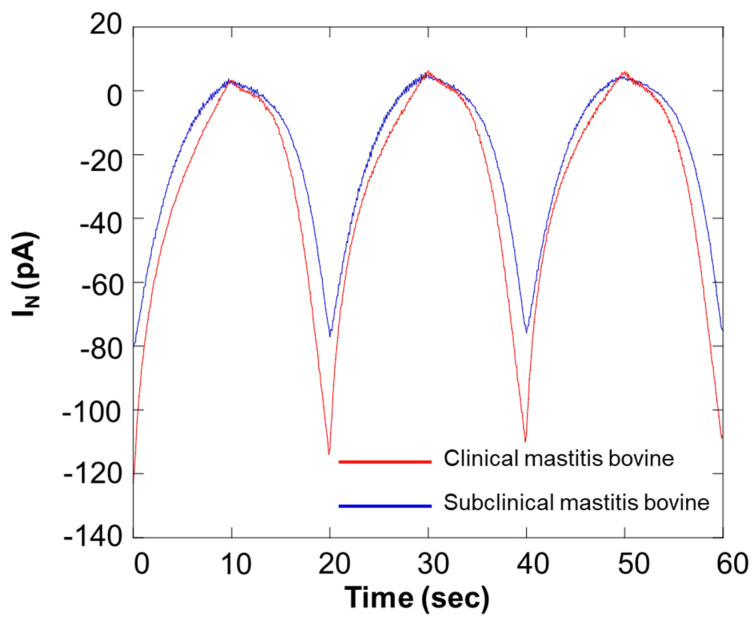
Comparison of respiration activity between bovines diagnosed with different stages of mastitis. I_N_: relative value of oxygen reduction current in bulk, which was set at 0 nA.

Figure 12 shows the results of an SECM-SCC study of milk samples. In the graph presented in Figure 12, the red and blue lines show the data from bovines diagnosed with different stage of mastitis. Because of scanning the microelectrode with SECM-SCC, notable differences were obtained at 20 s, 40 s, and 60 s. Table 3 shows the results of estimation of the number of somatic cells in the sample from the calibration curve in Figure 10.

## 4. Conclusions

Our results clearly show that the number of somatic cells can be measured by the evaluation of the respiratory activity. Because the degree of oxygen depletion is directly proportional to the number of cells, we can derive the number of cells in the milk. The chip device designed specifically for the dairy industry to be used at the farm was developed with the aim that it could serve as an effective method to access the quality of milk and/or to detect early onset of mastitis in buffalos, ensuring accuracy and cost effectiveness. The current assay suffers limitations due to the fact that it detects only live cells, and this can eventually lead to misuse of the technique and/or misinterpretation of results. Thus, the technique needs further development to pave the way to wider application.

## Figures and Tables

**Table 1:** Evaluation of somatic cells with inverted cone-shaped wells. The oxygen reduction current value difference between bulk and surface (ΔI) from Figure 3. The difference in oxygen reduction current was calculated using values at bulk (−1.76 nA) and oxygen concentration (248 μM).

Cell Number (cells/mL)	|ΔI| (pA)	ΔC (μM)
6.00 × 10^6^	105	15
6.00 × 10^5^	20	2.9

**Table 2:** SECM-SCC with the milk cell chip. The respiration rate (F) is calculated using Equation (1).

Number of Cells (cells/mL)	|ΔI| (pA)	ΔC (μM)	F × 10^14^ (mol/s/well)
1.10 × 10^6^	116	17	2.3
5.50 × 10^5^	70	10	1.4
5.50 × 10^4^	23	3.4	0.44

**Table 3:** Comparison of bovines by SECM-SCC. The number of somatic cells was estimated from Figure 10.

	|ΔI| (pA)	Number of Somatic Cells Estimated Using SECM-SCC (cells/mL)
Bovine sample 1	115	1.08 × 10^6^
Bovine sample 2	74	6.20 × 10^5^

## Data Availability

Not applicable.

## Supplementary Materials

The following supporting information can be downloaded at: https://www.mdpi.com/article/10.3390/biology11040549/s1, Figure S1: The oxygen reduction current from somatic cells in the milk cell chip filtered using nanofibers and without centrifugation. The raw milk determined using flow cytometry at Livestock improvement association of Japan. Approximately 0.27 g of nanofibers was inserted into a 10 mL disposable syringe and 5 mL milk (6.5 × 10^6^ cells/mL) was filtered through the syringe. SECM-SCC was performed in the same manner as in Section 2.5.
