# Supplementary material for: Scanning Electrochemical Microscopy-Somatic Cell Count as a Method for Diagnosis of Bovine Mastitis
Source: Biology (Basel). 2022 Apr 1;11(4):549. doi: 10.3390/biology11040549 (PMC9031417; doi:10.3390/biology11040549)
Supplement: Supplementary file 1 [file biology-11-00549-s001.zip › biology-1631656-supplementary.pdf]

Figure S1

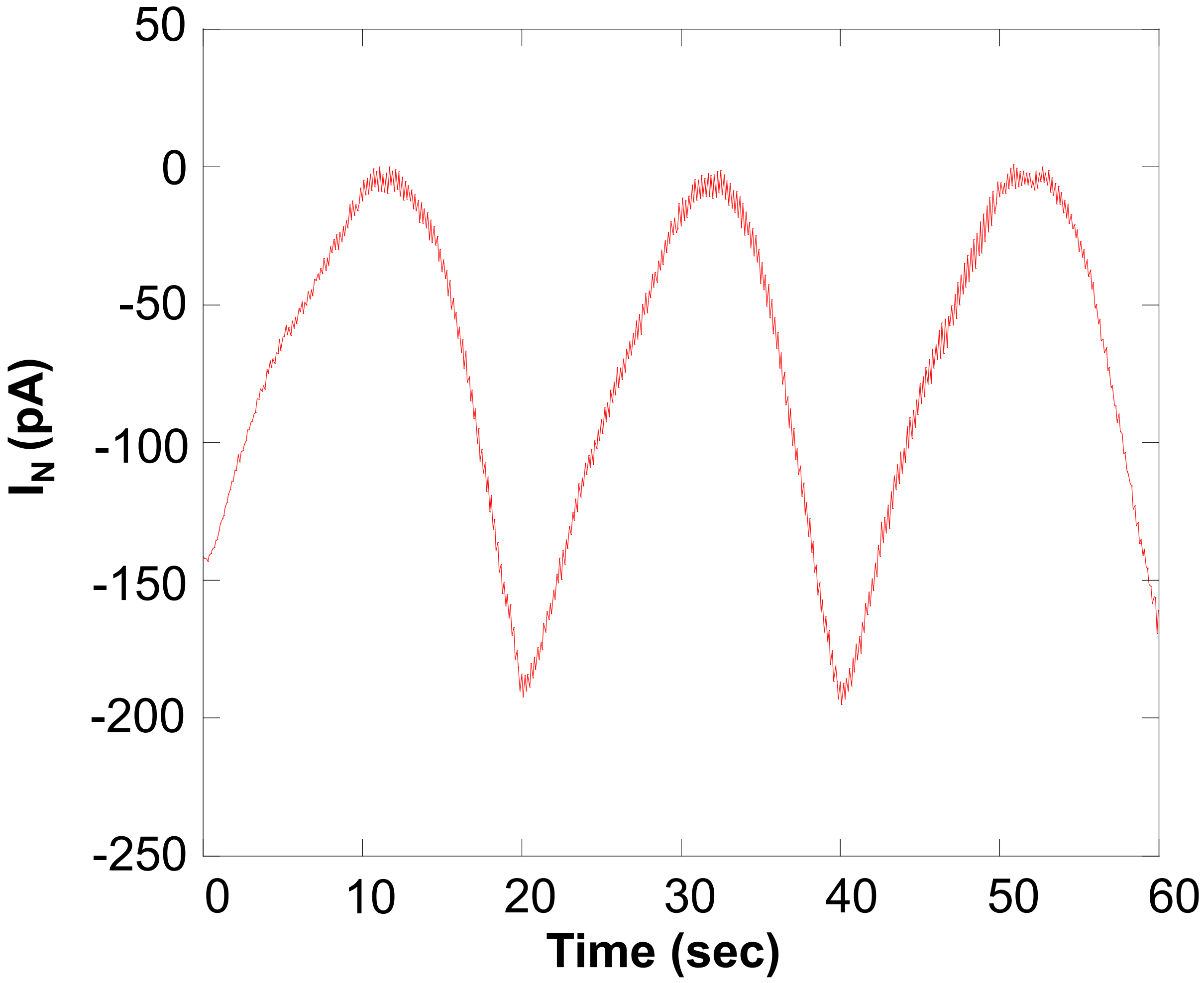

Figure S1: The oxygen reduction current from somatic cells in the milk cell chip filtered using nanofibers and without centrifugation. Approximately 0.27 g of nanofibers was inserted into a 10 mL disposable syringe and 5 mL milk (6.5×10^6 cells/mL) was filtered through a syringe. SECM-SCC was performed in the same manner as in section 2.5.
